# Dynamics of Tumor Hypoxia in Response to Patupilone and Ionizing Radiation

**DOI:** 10.1371/journal.pone.0051476

**Published:** 2012-12-10

**Authors:** Katrin Orlowski, Carla Rohrer Bley, Martina Zimmermann, Van Vuong, Daniel Hug, Alex Soltermann, Angela Broggini-Tenzer, Martin Pruschy

**Affiliations:** 1 Department of Radiation Oncology, University Hospital Zurich, Zurich, Switzerland; 2 Division of Radiation Oncology, Vetsuisse Faculty, University of Zurich, Zurich, Switzerland; 3 Department of Pathology, University Hospital Zurich, Zurich, Switzerland; 4 KFSP Tumor Oxygenation, University of Zurich, Switzerland; University Health Network, Canada

## Abstract

Tumor hypoxia is one of the most important parameters that determines treatment sensitivity and is mainly due to insufficient tumor angiogenesis. However, the local oxygen concentration in a tumor can also be shifted in response to different treatment modalities such as cytotoxic agents or ionizing radiation. Thus, combined treatment modalities including microtubule stabilizing agents could create an additional challenge for an effective treatment response due to treatment-induced shifts in tumor oxygenation. Tumor hypoxia was probed over a prolonged observation period in response to treatment with different cytotoxic agents, using a non-invasive bioluminescent ODD-Luc reporter system, in which part of the oxygen-dependent degradation (ODD) domain of HIF-1α is fused to luciferase. As demonstrated *in vitro*, this system not only detects hypoxia at an ambient oxygen concentration of 1% O_2_, but also discriminates low oxygen concentrations in the range from 0.2 to 1% O_2_. Treatment of A549 lung adenocarcinoma-derived tumor xenografts with the microtubule stabilizing agent patupilone resulted in a prolonged increase in tumor hypoxia, which could be used as marker for its antitumoral treatment response, while irradiation did not induce detectable changes in tumor hypoxia. Furthermore, despite patupilone-induced hypoxia, the potency of ionizing radiation (IR) was not reduced as part of a concomitant or adjuvant combined treatment modality.

## Introduction

Hypoxia is one of the most important parameters that cause enhanced tumor aggressiveness and treatment resistance, and hypoxia is now considered to be an independent prognostic indicator of poor outcome for different tumor entities. Alternating periods of hypoxia and normoxia in the tumor support the selection of tumor cells with elevated mutation frequency with a more stress resistant and aggressive phenotype. Independent of the cellular genotype, hypoxic cells are more treatment resistant than normoxic cells, in particular towards ionizing radiation (IR). Irradiation of cells leads to the formation of reactive oxygen species (ROS), which induce cytotoxic DNA damage. Furthermore the oxygenation fixation theory implies that radiation-induced free radical sites in the DNA are chemically derivatized (“fixed”) in the presence of oxygen so that they can not be repaired and accumulate, leading to an enhanced rate of cell death. Thereby, normoxic cells are two- to three-fold more radiation sensitive than cells under hypoxia [1], [2].

Tumor hypoxia is mainly caused by insufficient tumor angiogenesis and oxygen supply during tumor growth, however, the oxygen content in a tumor can also be shifted in response to different treatment modalities such as cytotoxic agents acting on the tumor vasculature. Therefore, the combination of cytotoxic agents, provoking an increase in tumor hypoxia, with ionizing irradiation may impact treatment efficiency. We previously investigated various combined treatment modalities with regard to changes in tumor hypoxia, e.g. VEGF-receptor tyrosine kinase inhibitors in combination with IR [3], [4]. Furthermore, the tumor- and tumor vasculature targeting, clinically relevant microtubule stabilizing agent (MSA) patupilone (epothilone B) induced an at least additive antitumoral effect when combined with IR [5], [6] raising the question on the dynamics of patupilone-induced hypoxia and the combination scheduling with IR.

MSAs belong to the most important classes of anti-cancer agents with taxanes being approved for a broad range of indications including single treatment for non-small cell lung carcinoma or advanced breast cancer [7], [8]. The epothilones are nontaxoid macrolide MSAs of bacterial origin, which share the same binding site on β-tubulin (in close proximity to residue Thr274) with taxanes [9], [10], [11]. Clinically different epothilone derivatives are currently in various stages of development as antitumor compounds [12]. Ixabepilone (Ixempra®) is the first approved compound in this class and indicated as monotherapy or in combination with capecitabine for the treatment of patients with metastatic breast cancer. Apart from a manageable safety profile, ixabepilone demonstrates anti-tumor activity after failure and resistance towards anthracycline and taxane standard therapy [13].

Epothilone B (patupilone) was tested as a phase III monotherapy agent against ovarian cancer and other epothilones are undergoing a wide spectrum of single and combined treatment modality in phase II studies (e.g. for recurrent glioblastoma, CNS metastases from breast cancer, prostate, cervical, renal cell, gastric and lung tumors, as well as non-Hodgkin’s Lymphoma (www.cancer.gov) [13], [14], [15], [16], [17].

MSAs impair the dynamics of the microtubule network, leading to defective mitotic spindle formation and accumulation of cells in the G2/M-phase of the cell cycle [11], [18] or at low concentrations to transient G1- and S-phase arrest [5], [19], followed by apoptosis-induction [20]. An MSA-altered microtubule network also reduces the cellular migration and invasion capacity [21], [22]. Furthermore, the potential of MSAs to accumulate cells in the radiosensitive G2/M phase renders them potent sensitizers [23] for the combined treatment with ionizing radiation [5], [6], [24].

The hypoxia-inducible transcription factor HIF-1 is a heterodimer composed of an oxygen-sensitive alpha subunit and a constitutively expressed beta subunit. HIF-1 binds to the hypoxia response element (HRE) in the promoter region of diverse target genes such as VEGF and induces their expression [25]. Under normoxic conditions the alpha subunit is hydroxylated on proline402 and proline564 in the oxygen-dependent degradation (ODD)-domain of HIF-1α by prolyl hydroxylase domain (PHD) proteins. Proline hydroxylation leads to the recognition by the von Hippel-Lindau tumor suppressor and subsequent ubiquitination and proteasomal degradation [26].

Tumor hypoxia has previously been probed using different luciferase-based bioimaging reporter constructs, which are either based on luciferase expression under the control of an HRE-based promoter system or on the fusion of extended or shorter ODD-domains to the reporter gene [27], [28], [29]. The HRE-based approach depends on intact HIF-signaling, however, several classes of antisignaling agents including microtubule stabilizing agents interfere with the activity of HIF-1-upstream elements or the direct expression of HIF-1, independent of the pO_2_ [6], [30], [31], [32], [33], [34]. We therefore did not use an HRE-based but a minimal ODD-based *in vivo* bioimaging reporter approach, which demonstrated high sensitivity even in the range of low pO_2_-levels, to serially probe the dynamics of tumor hypoxia in response and in relation to the antitumor effect of the microtubule stabilizing agent patupilone and ionizing radiation, alone and as part of the combined treatment modality.

## Materials and Methods

### Cell Culture

The human colon carcinoma cell line HCT116 was obtained from Bert Vogelstein [35] and the lung adenocarcinoma cell line A549 from Susan Band Horwitz [36]. All cell lines were kept at 37°C in 5% CO_2_. The cell line HCT116 was grown in McCoy medium containing 10% (v/v) fetal bovine serum, 100 U/ml penicillin and 100 µg/ml streptomycin. The cell line A549 was grown in RPMI 1640 containing 10% (v/v) fetal bovine serum, 100 U/ml penicillin, 100 µg/ml streptomycin and 2 mM L-glutamine. Patupilone (epothilone B, EPO906) was provided by the chemistry department of Novartis Pharma AG (Basel, Switzerland).

### Vector Construction

The plasmid SV40-pGL4.27 was obtained by inserting the SV40 promoter of pGL3control (Promega Corporation, Madison, WI, USA), cut with the restriction enzymes KpnI and HindIII, into the pGL4.27 vector (Promega Corporation) containing the luciferase gene including a 3′PEST sequence for rapid degradation and turnover of the luciferase protein. An additional NarI restriction site was inserted in the pGL4.27 plasmid at position 174 by site directed mutagenesis. The ODD-domain of HIF-1α was amplified by PCR from the pcDHIF plasmid (kindly provided by R.Wenger) as described in Safran et al, 2005 [37]. The product was cloned into the pGL3 basic vector (by HindIII and NarI) and finally inserted into the SV40-pGL4.27 vector, containing the additional NarI-site, to obtain the vector construct SV40-ODD-pGL4.27. To obtain the plasmids SV40-pGL4.26 and SV40-ODD-pGL4.26, the SV40 promoter or the SV40-ODD sequence was subcloned from the respective pGL4.27 plasmids cut by KpnI and BsrG1 into the pGL4.26 backbone (Promega Corporation).

### Stable Transfection, Reporter Gene Assay and Western Blot Analysis

HCT116 cells were stably transfected with the SV40-ODD-pGL4.27 plasmid and the A549 cells were stably transfected with the SV40-ODD-pGL4.26 or the SV40-pGL4.26 plasmid by lipofection (Lipofectamine™ 2000 system; Invitrogen, Carlsbad, CA, USA) following the manufacturer’s instruction. Stable single clones from hygromycin selected pools were selected for the highest induction of luciferase activity after 8 hours incubation under hypoxic conditions. The reporter gene assay was performed as described by Rohrer Bley et al, 2009 [6]. Hypoxic conditions were mimicked by the prolyl hydroxylase inhibitor dimethyloxalylglycine (DMOG) (Biomol GmbH, Hamburg, Germany) at a concentration of 0.25 mM or CoCl_2_ (0.25 mM). Alternatively, cells were incubated under different pO_2_ in the hypoxic chamber (Invivo2 400 hypoxia workstation, Biotrace International, Bridgend, UK). Cells were allowed to attach for 8–10 hours before addition of patupilone. Hypoxic conditions were applied 24 hours after patupilone treatment and luciferase activity was determined 8 hours thereafter. Western blot analyses were performed as described by Rohrer Bley et al, 2011 [38].

### Clonogenic Cell Survival Assay

Clonogenic survival was determined by the ability of single cells to form colonies *in vitro*. The number of plated cells was adjusted to obtain ∼100 colonies per cell culture dish with a given treatment. After treatment with the different regimes, the dishes were maintained at 37° in 5% CO_2_ and allowed to grow for 12 days before fixation in methanol/acetic acid (3∶1) and staining with crystal violet. Cells were either preincubated with patupilone for 18 hours followed by irradiation, or preincubated with patupilone for 18 hours followed by 7 days of cultivation in new media, reseeded and irradiated on day 9, or preincubated with patupilone for 8 days (in patupilone containing media), reseeded and irradiated on day 9. Colonies with >50 cells/colony were counted manually. All assays were repeated as independent experiments at least thrice.

### Hypoxyprobe-1 Immunofluorescence

A549 cells were seeded on glass cover slips and allowed to attach overnight. Medium was exchanged and cells were incubated with 150 µM hypoxyprobe-1 (HPI, Burlington, MA, USA) for 3.5 hours under normoxic or hypoxic conditions. Culture slides were washed with phosphate buffered saline (PBS), fixed in 4% formaldehyde for 10 minutes at RT. Cells were washed and permeabilized with 0.2% Triton X-100 buffer on ice for 10 minutes. Cells were washed and autofluorescence was quenched with 0.3 M glycin for 5 minutes. After preincubation with 3% BSA in PBS at RT for 30 minutes, cells were incubated with the fluorescein isothiocyanate (FITC)-labeled monoclonal anti-Hypoxyprobe-1 monoclonal antibody (mAb1) in 3% BSA/PBS, (HPI), at RT in the dark for 1 hour. DNA was counterstained with DAPI (1∶2500 DAPI stock solution (1 mg/ml) in PBS Sigma) for 15 minutes at RT. Cells were finally washed with PBS. Images were captured by fluorescence microscopy using a CCD camera (Leica DM6000B equipped with Leica CTR6000).

### Tumor Xenograft in Nude Mice and Application of Treatment Regimes

Stably transfected HCT116 cells (4×10^6^) and A549 cells (7×10^6^) were subcutaneously injected on the back of 4- to 8-week old athymic nude mice. Tumors were allowed to expand to a volume of 300 mm^3^ (±10%) or 200 mm^3^ (±10%) before treatment start. Tumor volumes were determined as described elsewhere [6]. The group sizes ranged from n = 7–12 for the HCT116-xenograft experiments, from n = 3–5 for the A549-Luc only and CoCl_2_-control experiments, and n = 5–16 for all other experiments. Patupilone (dissolved in 30% PEG-300/70% saline) was applied i.v. at a concentration of 2 mg/kg and CoCl_2_ (45 mg/kg, dissolved in saline) i.p. after the first IVIS measurement (day 0). Control mice were treated i.v. or i.p. with saline. IVIS measurements were performed daily at the indicated time points. Fractionated irradiation (3×1 Gy on 3 consecutive days) was applied locally using a customized led shielding device with a Gulmay 200 kV X-ray unit at 1 Gy/min.

### Statement of Ethical Approval

This study was performed in strict accordance with the recommendations in the Guide for the Care and Use of Laboratory Animals of the Swiss Cantonal Veterinary Authorities. The protocol was approved by the Committee of the Swiss Cantonal Veterinary Authorities (Permit Number: 136/2008 and 154/2011). All procedures and measurements were performed under isoflurane anesthesia, and every effort was made to minimize suffering.

### *In vivo* Bioluminescence Imaging and Analysis

Mice were i.p. injected with 150 mg/kg D-luciferin (CaliperLife Sciences, Hopkinton, MA, USA) (10 µl/g of a 15 mg/ml stock solution) prior to anesthesia. Sequential measurements of light emission (total flux) were taken approx. 5 minutes after D-luciferin injection with the IVIS200 (CaliperLife Sciences). The measurement with the highest total flux in the respective region of interest (ROI), within the sequential measurement of each day and mouse, was used for the longitudinal survey. The values were normalized to day 0 and divided by the tumor volume to correct for treatment-dependent changes.

### Statistical Analysis

*In vitro* data presented are representatives of three or more independent experiments, if not otherwise indicated. Student’s t-test was used to evaluate the differences between normoxic and hypoxic (incl. DMOG and CoCl_2_) conditions. Statistical analysis of the *in vivo* tumor growth and luciferase activity data was performed with the Mann-Whitney U test. The relative tumor growth delay and the fold induction of luciferase activity/tumor volume were normalized to day 0. The level of significance was set at 0.05; the calculations were all performed using the GraphPad Prism software version 5 (GraphPad Software Inc.).

## Results

### Establishment and Evaluation of a Highly Sensitive *in vitro* and *in vivo* Tumor Hypoxia Reporter System

**Figure 1:**
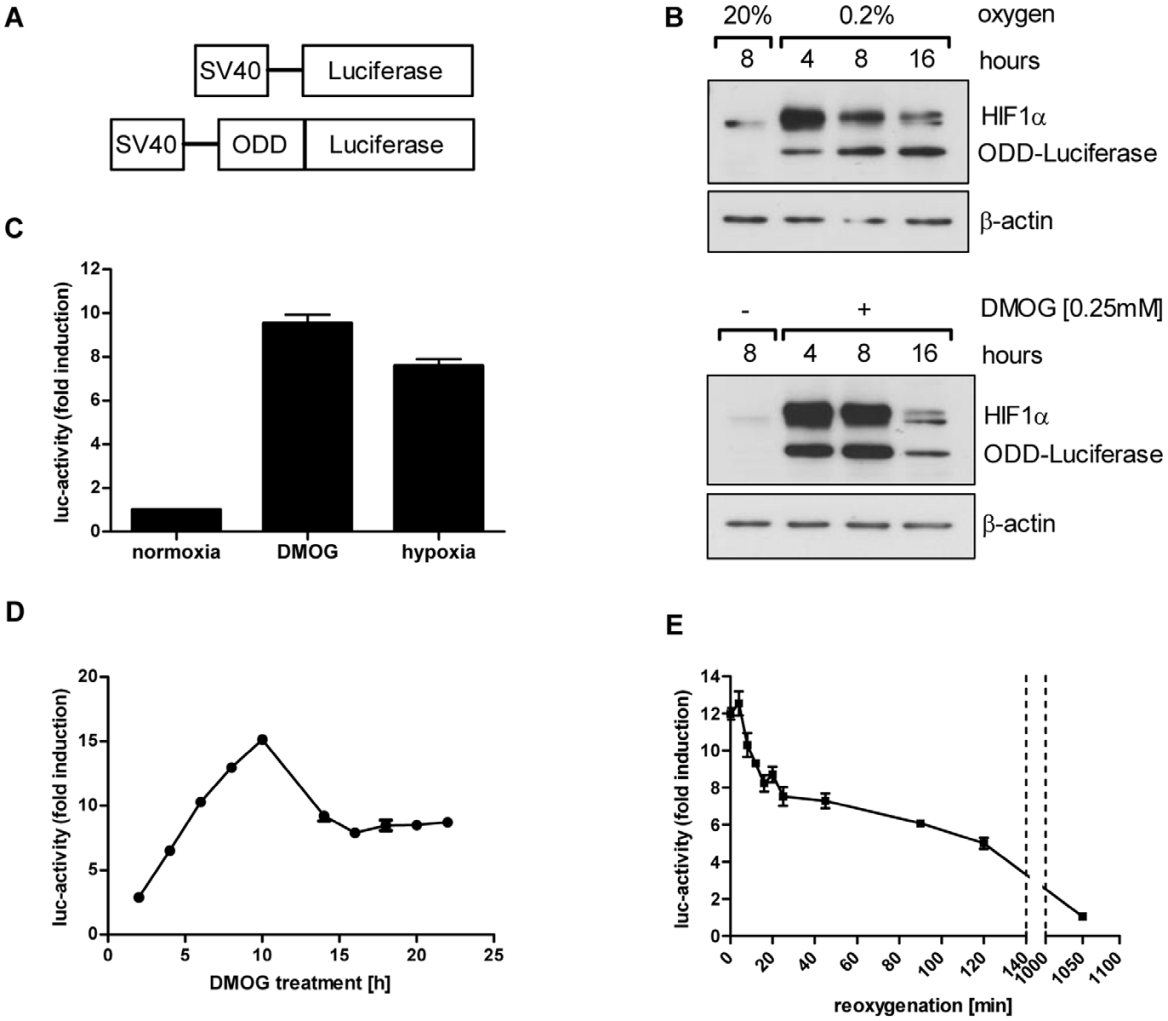
Oxygen-sensitive luciferase reporter system. (A) Scheme of luciferase constructs for constitutive (Luc) and oxygen-sensitive luciferase expression (ODD-Luc), containing a part of the oxygen-dependent degradation (ODD) domain of HIF-1α. Both constructs are under the control of a minimal SV40-promoter. (B) Protein levels of HIF-1α and ODD-luciferase in cellular extracts of stably transfected A549 cells, incubated for different time periods under normoxia, 0.2% O_2_ or DMOG. (C) Luciferase activity in stably transfected ODD-Luc A549 cells. Cells were incubated for 8 hours under normoxia, DMOG or hypoxia. (D) Luciferase activity in DMOG-treated, stably transfected ODD-Luc A549 cells over a 22 hour period. (E) Luciferase activity in stably transfected ODD-Luc A549 cells kept for 8 hours under hypoxia and reoxygenated (time point 0 minutes) thereafter. Error bars represent mean ±SE.

To monitor changes in tumor hypoxia *in vivo*, a reporter gene system was constructed, which consists of an oxygen-dependent degradation (ODD) domain fused 5′ to the luciferase reporter gene (ODD-Luc). This construct is constantly expressed in cells under control of a minimal, hypoxia-independent, SV40-promoter to be rapidly degraded under normoxic conditions, and slightly differs from previously used ODD-based constructs [29], [39]. The ODD-sequence derives from the originally-identified human oxygen-dependent degradation domain (ODD) of HIF-1α but includes only the sequence coding for aa530 to aa652. This shorter ODD includes proline564, which is hydroxylated under normoxia and thereby marked for ubiquitin-dependent degradation by the VHL-proteasome-pathway, but excludes the NO-sensitive Cys-residue at amino acid position 520 (corresponding to mouse HIF-1α aa533), which could lead to a hypoxia-independent stabilization of the reporter construct in the presence of tumor associated macrophages [39]. A luciferase reporter construct without an ODD and under control of the same SV40-promoter served as control system (Figure 1A).

Hypoxia-sensitivity of the ODD-luciferase reporter construct was probed in stably-transfected A549 non-small cell lung cancer cells under hypoxia and hypoxia-mimicking conditions using the specific prolyl hydroxylase domain (PHD) inhibitor dimethyloxalylglycine (DMOG) at non-toxic conditions. Western blot analysis of cell lysates derived from cells incubated under normoxic and hypoxic conditions (0.2% O_2_) or with DMOG (0.25 mM) revealed high hypoxia- and DMOG-dependent protein levels of the ODD-luciferase construct. Prolonged expression level of the construct was observed under permanent hypoxic conditions but the ODD-luciferase expression level decreased over time in DMOG-treated cells (Figure 1B). This might be due to a reduced potency of DMOG to constantly inhibit related PHDs. HIF-1α protein stability paralleled ODD-luciferase protein levels under these conditions but also decreased over time, most probably due to a previously described regulatory feedback mechanism [40]. An *in vitro* luciferase activity assay also clearly demonstrated a significantly strong hypoxia and DMOG-dependent increase in luciferase activity in this stably transfected tumor cell line (Figure 1C, p<0.005).

The kinetics of ODD-luciferase activity was further investigated after cellular treatment with DMOG during a 22-hours time course experiment and demonstrated the highest fold-increase in ODD-luciferase activity 8–10 hours after PHD-inhibition, with a subsequent slow decrease of luciferase activity over time (Figure 1D). Keeping cells under hypoxia for 8 hours followed by full reoxygenation to aerated conditions resulted in a rapid decrease of luciferase activity within the first 20 minutes, to reach 50% of its activity under hypoxic conditions already 90 minutes after reoxygenation (Figure 1E).

**Figure 2:**
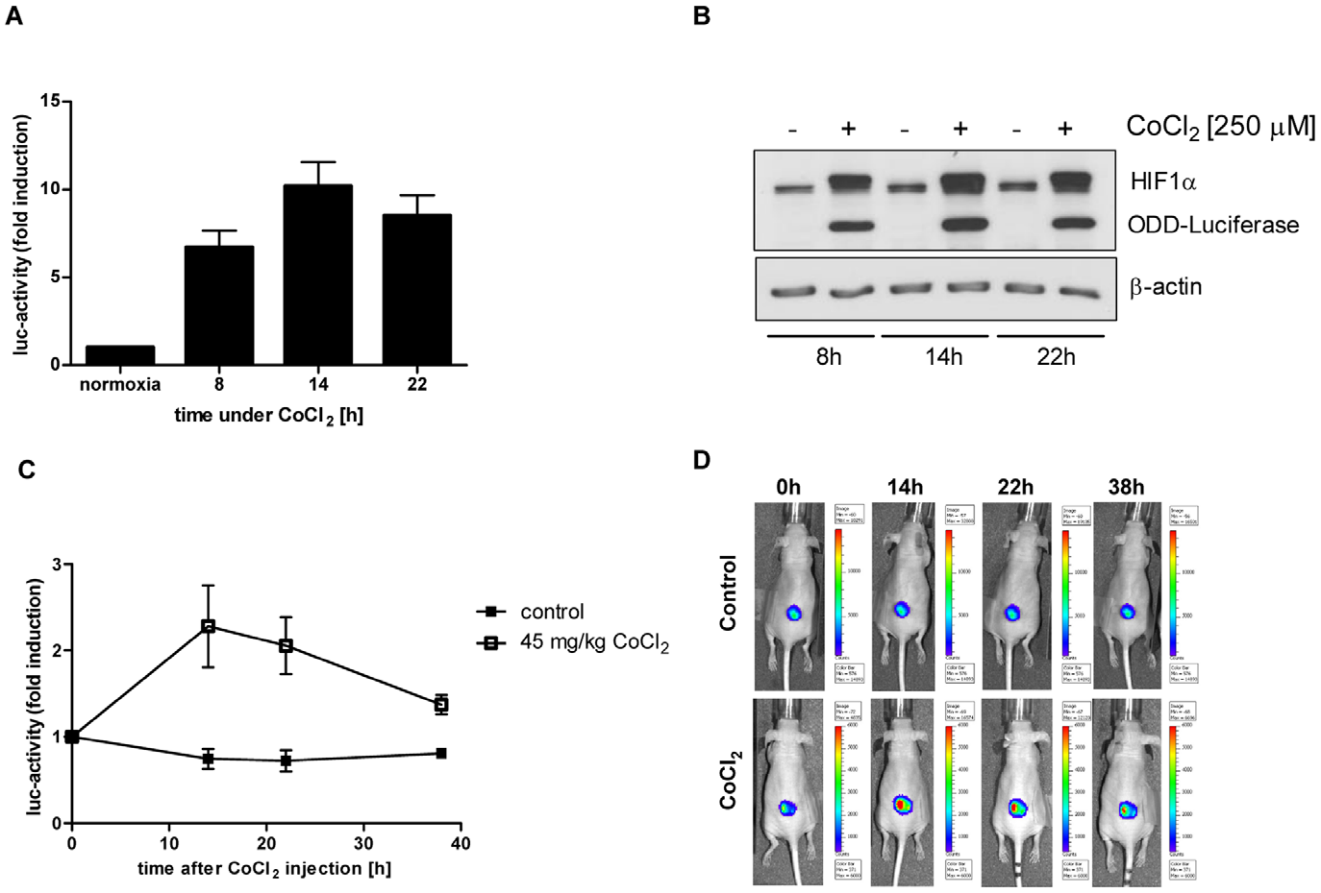
CoCl_2_ increases luciferase activity in A549 ODD-Luc cells *in vitro* and *in vivo*. (A,B) Luciferase-activity (A) and HIF-1α, and protein levels of ODD-luciferase (B) of CoCl_2_ (0.25 mM)-treated, stably transfected ODD-Luc A549 cells, determined at the indicated time points. Error bars represent mean ±SE. (C) Luciferase activity of A549 ODD-Luc-derived tumor xenografts in mice injected with CoCl_2_ (45 mg/kg) at time point 0 hour and at the indicated time points thereafter. (n_CoCl2_ = 6; n_control_ = 4) Error bars represent mean ±SE. (D) Representative *in* *vivo* bioimages of untreated and CoCl_2_-treated mice.

To validate this non-invasive ODD-luciferase reporter system *in vivo*, we used cobalt salt CoCl_2_, which also inhibits PHDs. CoCl_2_ (0.25 mM) induced a time-dependent increase in HIF-1α and ODD-luciferase protein levels *in vitro* (Figure 2B), and cellular luciferase activity peaked 14 hours after addition of CoCl_2_ (Figure 2A, p<0.007). Mice carrying tumor xenografts, which derived from ODD-Luc stably transfected A549 cells, were treated with CoCl_2_ (45 mg/kg, i.p.), and luciferase activity in the tumor xenograft was determined 14, 22 and 38 hours after CoCl_2_-treatment by *in vivo* bioimaging. Luciferase activity significantly increased in the tumor xenografts after CoCl_2_-treatment, reached a maximum of 2.5-fold induction at the 14 hour time point after treatment (p<0.05) and returned to basal levels 38 hours after treatment start. In comparison, luciferase activity in placebo-treated control mice did not change over this 38 hour period (Figure 2C and D).

**Figure 3:**
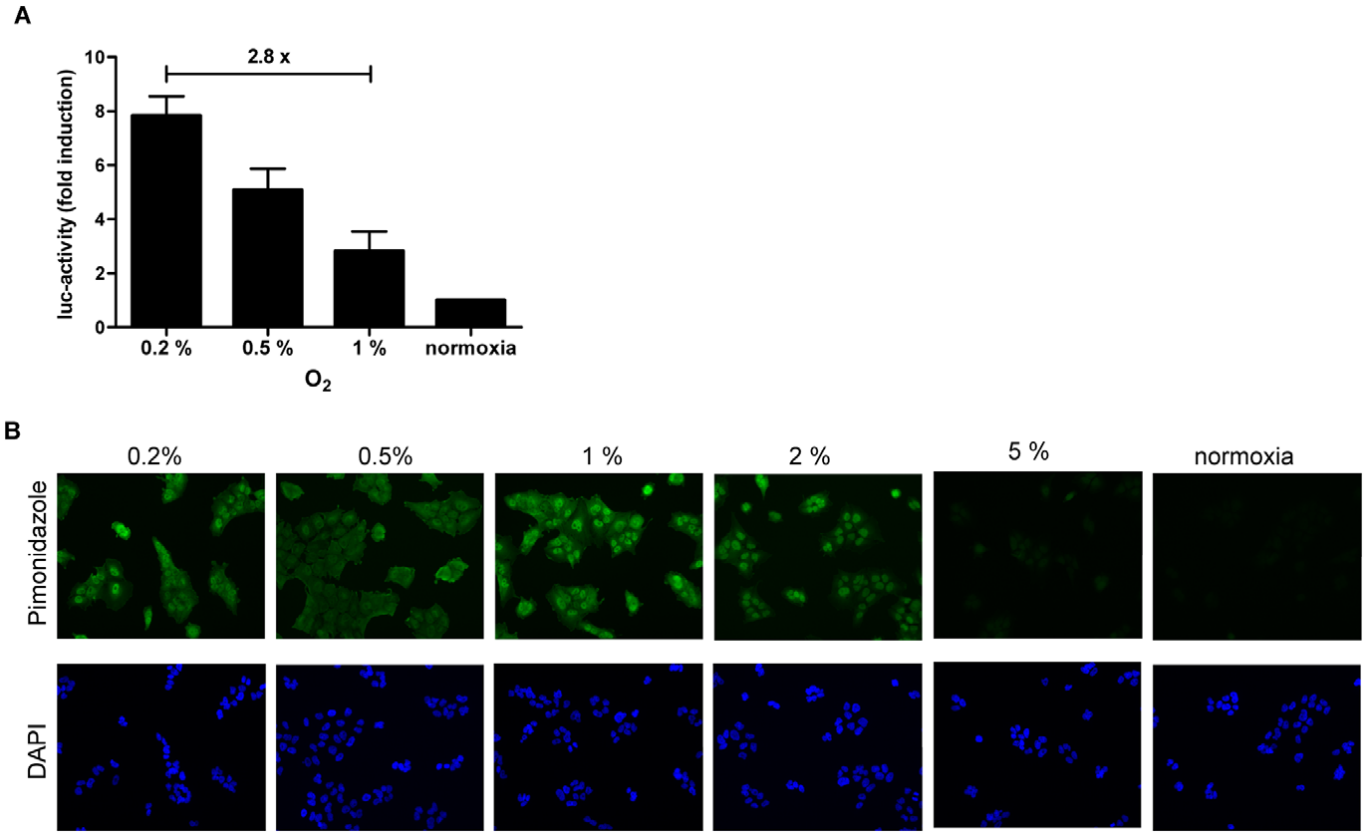
Differential sensitivity for low levels of pO_2_ by ODD-luciferase and pimonidazole. (A) Luciferase activity in A549 ODD-Luc cells was determined after cellular incubation for 8 hours at different levels of hypoxia and normoxia. Error bars represent mean ±SE. (B) Detection of pimonidazole accumulation in A549 cells incubated at different levels of hypoxia and normoxia. Images display immunocytochemistry staining of pimonidazole and DAPI for nuclear staining (magnification, ×300).

To determine the sensitivity of the luciferase reporter system towards different levels of hypoxia, stably-transfected A549 cells were incubated for 8 hours at 0.2, 0.5, 1 and 2% O_2_. Interestingly, the luciferase activity constantly increased with cellular incubation at lower pO_2_-levels. An 8-fold increase in luciferase activity from normoxia to 0.2% O_2_ and a 2.8-fold increase in luciferase activity from 1% to 0.2% O_2_ was observed (p<0.01), which indicate a high sensitivity towards different levels of hypoxia (Figure 3A).

The bioreductive hypoxia marker pimonidazole (Hypoxyprobe-1) forms stable adducts with thiol (sulphydryl) groups in proteins below the threshold of 10 mmHg pO_2_, corresponding to approx. 1.5–2% O_2_. To analyze whether different low levels of pO_2_ can be distinguished from each other by an increased cellular accumulation of pimonidazole, A549 cells were incubated with pimonidazole (150 µM) under normoxia and various concentrations of O_2_ (0.2, 0.5, 1, 2 and 5% O_2_) for 3.5 hours and stained with an FITC-labeled anti-pimonidazole antibody. The staining intensity of pimonidazole increased in a dose dependent way from normoxia to 2% O_2_, however no difference in the staining intensity could be detected below 1% O_2_ (Figure 3B).

Overall these *in vitro* and *in vivo* results obtained with the non-invasive ODD-Luc-reporter approach demonstrate the high practicability and sensitivity to monitor dynamic changes in tumor hypoxia *in vitro* and *in vivo*.

### Changes in Tumor Hypoxia as Marker for Treatment Response to Cytotoxic Agents

**Figure 4:**
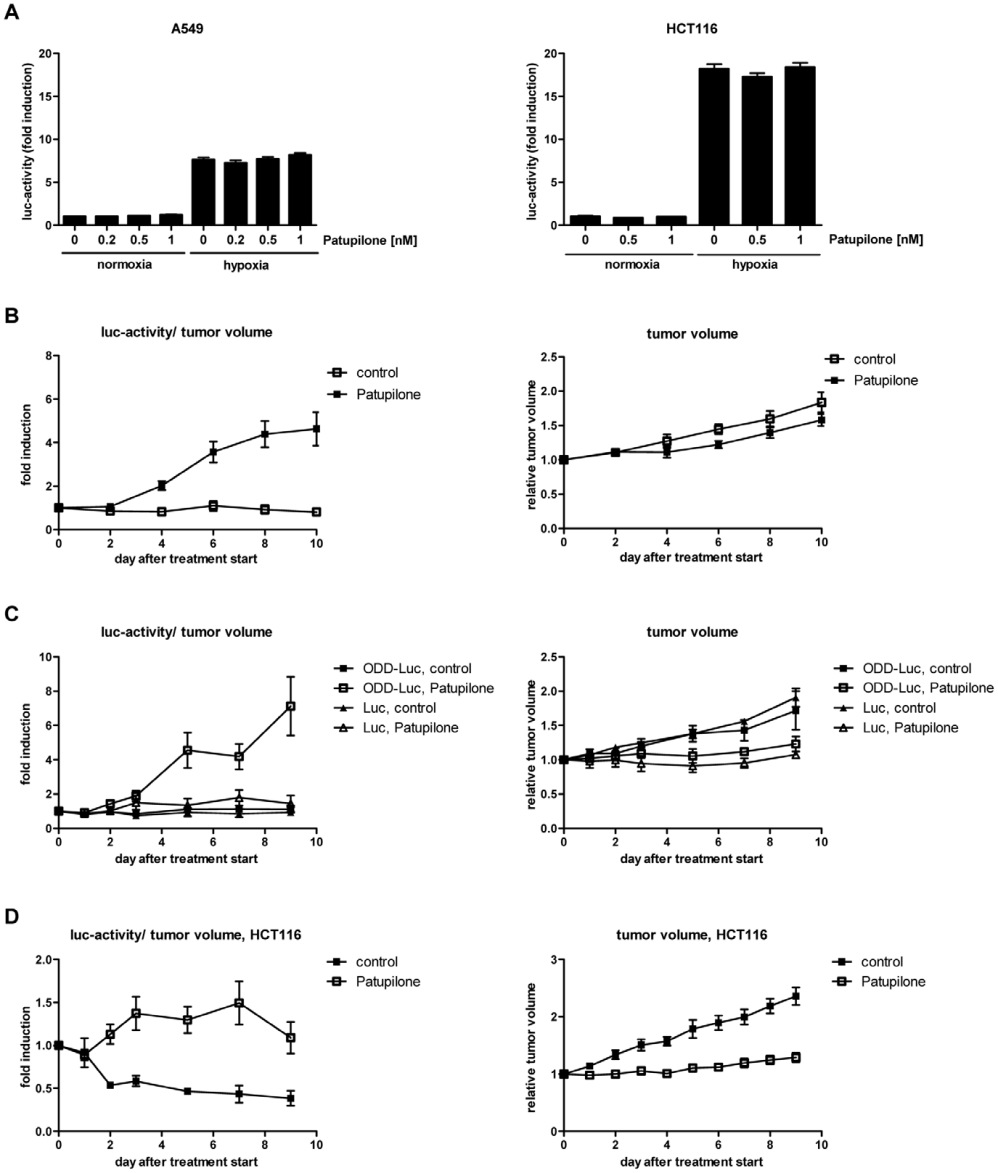
Patupilone-dependent increase in tumor hypoxia *in vivo*. (A) Stably-transfected A549 ODD-Luc and HCT116 ODD-Luc cells were treated with increasing concentrations of patupilone for 24 hours before incubation under normoxia and hypoxia (0.2% O_2_). Luciferase activity was determined after 8 hours of hypoxia. Error bars represent mean ±SE. (B) Luciferase activity (left side) and tumor growth (right side) of A549 ODD-Luc-derived tumor xenografts in mice treated with patupilone (2 mg/kg). (n_control_ = 7; n_patupilone_ = 19) (C) Luciferase activity and tumor growth of A549 ODD-Luc and A549 Luc-only-derived xenografts in mice treated with patupilone (2 mg/kg). (n_ODD-Luc_ = 5; n_Luc_ = 3) (D) Luciferase activity and tumor growth of HCT116 ODD-Luc-derived xenografts in mice treated with patupilone (2 mg/kg). (n_control_ = 7; n_patupilone_ = 12) Error bars represent mean ±SE of fold induction luciferase activity/tumor volume or relative tumor volume per group.

Using logistically-demanding non-invasive small animal positron emission tomography and classic invasive immunohistochemical approaches, we previously demonstrated that cytotoxic, anti-signaling and anti-angiogenic agents affect the tumor microenvironment and thereby tumor hypoxia [3]. In the intracellular signaling-based bioluminescence reporter system used in this report, which has been designed to investigate the dynamics of tumor hypoxia under different treatment modalities, the level of reporter gene expression and activity must be virtually independent of direct interference with the agent of interest (see introduction). To control for such a putative, detrimental interference, luciferase activity in the human A549 lung adenocarcinoma and the human HCT116 colon adenocarcinoma cell line, both stably transfected with the ODD-Luc-construct, was determined *in vitro* on treatment with increasing concentrations of patupilone under normoxic and hypoxic conditions. Hypoxia significantly increased luciferase activity in both cells lines (p<0.01) but cellular treatment with increasing concentrations of patupilone did not perturb luciferase activity significantly neither under normoxic nor hypoxic conditions (Figure 4A).

Having in hand a sensitive system to probe tumor hypoxia *in vivo,* the dynamics of tumor hypoxia in response to patupilone treatment was monitored over a prolonged observation period. Stably-transfected A549 ODD-Luc cells were subcutaneously injected on the back of nude mice and tumors were allowed to grow until a volume of 200 mm^3^+/−10%. Mice were then treated with patupilone as a single dose (2 mg/kg, i.v.) and luciferase activity in control and patupilone-treated mice was determined over 10 days by *in vivo* bioimaging. The basal level of luciferase activity in A549 ODD-Luc xenografts was measured prior to patupilone injection and treatment-dependent changes in luciferase activity were further corrected for treatment-dependent changes in the tumor volume (see Material and Methods). A significant increase in luciferase activity already occurred on day 4 in tumor xenografts of mice treated with patupilone in comparison to placebo-treated mice and reached a maximal induction of luciferase activity at day 10 after treatment (4.6-fold versus 0.8-fold in control tumors, p<0.001) (Figure 4B). Patupilone-treatment also significantly inhibited tumor growth during this observation period (Figure 4B). Relative luciferase activity declined approximately 20 days after treatment, in mice probed over a prolonged time period (data not shown). The ability of the ODD-Luc system to serially probe the luminescence in individual mice under treatment qualifies this system and demonstrates its robustness (Figure S1).

Control experiments were performed with tumor xenografts derived from A549 cells, which were stably-transfected with intact ODD-luciferase construct or a luciferase construct lacking an ODD-domain (Luc-only) (Figure S1A). With the latter construct, interference of patupilone with the SV40 promoter or with D-luciferin uptake and diffusion could be excluded. Tumors were allowed to grow until a volume of 200 mm^3^+/−10%, and mice were treated with either placebo or patupilone (2 mg/kg). Growth rate was similar for both A549 ODD-Luc and A549 Luc-only-derived tumor xenografts, and patupilone induced a comparable tumor growth delay for both tumor types. In contrast luciferase activity only increased after patupilone treatment in tumors derived from the ODD-Luc expressing A549 cells. These results further corroborate the specificity of the hypoxia-reporter construct to monitor treatment-related changes *in vivo* (Figure 4C).

To test for patupilone-increased tumor hypoxia in other tumor cell systems, the human colorectal adenocarcinoma cell line HCT116 was stably transfected with the ODD-Luc-construct and xenotransplanted in nude mice. HCT116 ODD-Luc derived tumors were allowed to grow to a volume of 300 mm^3^+/−10%, and mice were treated with placebo or a single dose of patupilone (2 mg/kg). Tumor growth was significantly inhibited (p<0.005) and luciferase activity also significantly increased in this tumor model in response to patupilone treatment (p<0.01; day 2–9). However, increase in luciferase activity already peaked on day 7 after patupilone treatment, though to a lesser extent in comparison to the A549-derived tumor system (Figure 4D). Histologic-morphologic analysis revealed a high percentage of necrosis (>50%) in this tumor model, thus complicating tumor volume standardization. Therefore, further experiments were only performed with the A549-derived tumor model with a low level of tumor necrosis.

**Figure 5:**
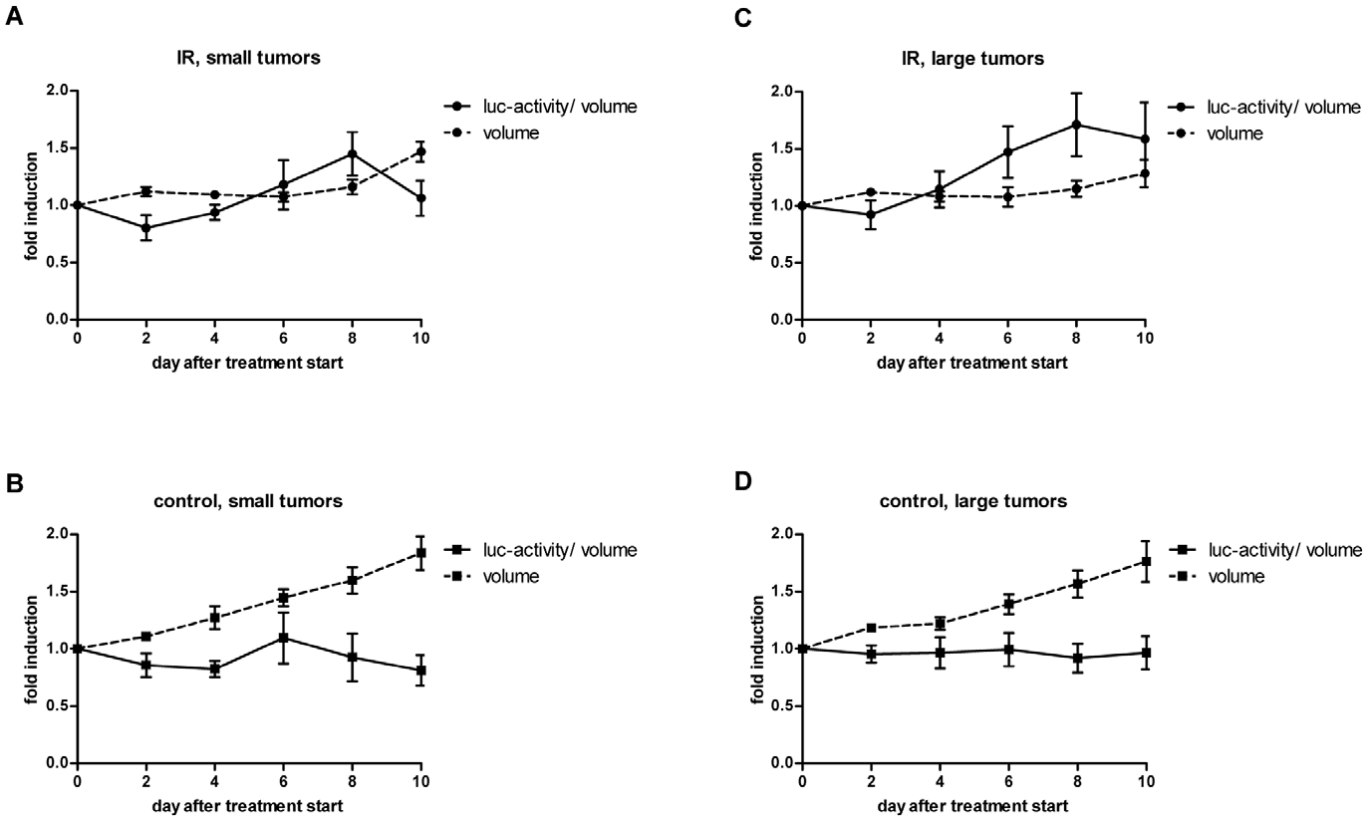
Minimal effect of ionizing irradiation on tumor hypoxia. Luciferase activity and tumor volume of A549 ODD-Luc-derived xenografts in control (B, D) and irradiated mice (A, C, 3×1 Gy) at a small (A, B, 200 mm^3^±10%) and at a large (C, D 400 mm^3^±10%) tumor volume at the day of treatment start (n_IR_ = 5; n_control_ = 7). Fold induction of luciferase activity per tumor volume (solid line) and relative tumor volume (dotted line) were set to 1 at day 0. Error bars represent mean ±SE of fold induction luciferase activity/tumor volume or relative tumor volume per group.

Tumor hypoxia is an important parameter for tumor radiosensitivity, and fluctuation of tumor hypoxia may influence the treatment response to radiotherapy. Control experiments with A549 ODD-Luc and A549 Luc-only cells performed *in vitro* under normoxia and hypoxia-mimicking conditions (DMOG) revealed that irradiation does not affect expression of these reporter construct per se. Likewise irradiation did not change luciferase activity in tumor xenografts expressing the Luc-only construct (Figure S2). Short-term fractionated irradiation was therefore used as a second cytotoxic treatment modality to probe the dynamics of tumor hypoxia under treatment (Figure 5). A549 ODD-Luc-derived tumor xenografts with a small or a large tumor volume (200 mm^3^ and 400 mm^3^, respectively) were irradiated with a minimally fractionated treatment regimen of 3×1 Gy on 3 consecutive days and luciferase activity was determined during treatment and seven follow-up days. In the two control groups covering small and large tumor volumes, tumors steadily increased in size and volume-corrected luciferase activity did not change over time (Figure 5B and D). The minimal irradiation regimen suppressed tumor growth until day 8 after treatment start in the group of small tumors, and during the entire observation period in the group with large tumors (Figure 5A and C). Irradiation induced a slight increase in luciferase activity in both groups (1.5 to 1.7-fold), which remained stable in the group of large tumors but again decreased to basal level in the group of small tumors towards the end of the observation period. Interestingly, this drop of volume-corrected luciferase activity coincided with resumed tumor growth and remained at basal levels during subsequent tumor growth (data not shown).

### Patupilone-induced Hypoxia does not Impair the Radiation Response

We previously investigated the combined treatment modality of patupilone and ionizing radiation and determined an at least additive treatment response on concurrent treatment on A549, SW480 colon carcinoma and D425Med medulloblastoma xenografts [5], [6], [41]. We now aimed to investigate the dynamics of tumor hypoxia, as indicated by changes in luciferase activity, in response to this combined treatment modality and to determine a putative counteractive effect of patupilone-induced tumor hypoxia on the radiation response. Therefore, A549 ODD-Luc-derived tumor xenografts were treated with different treatment schedules and luciferase-activity was monitored.

**Figure 6:**
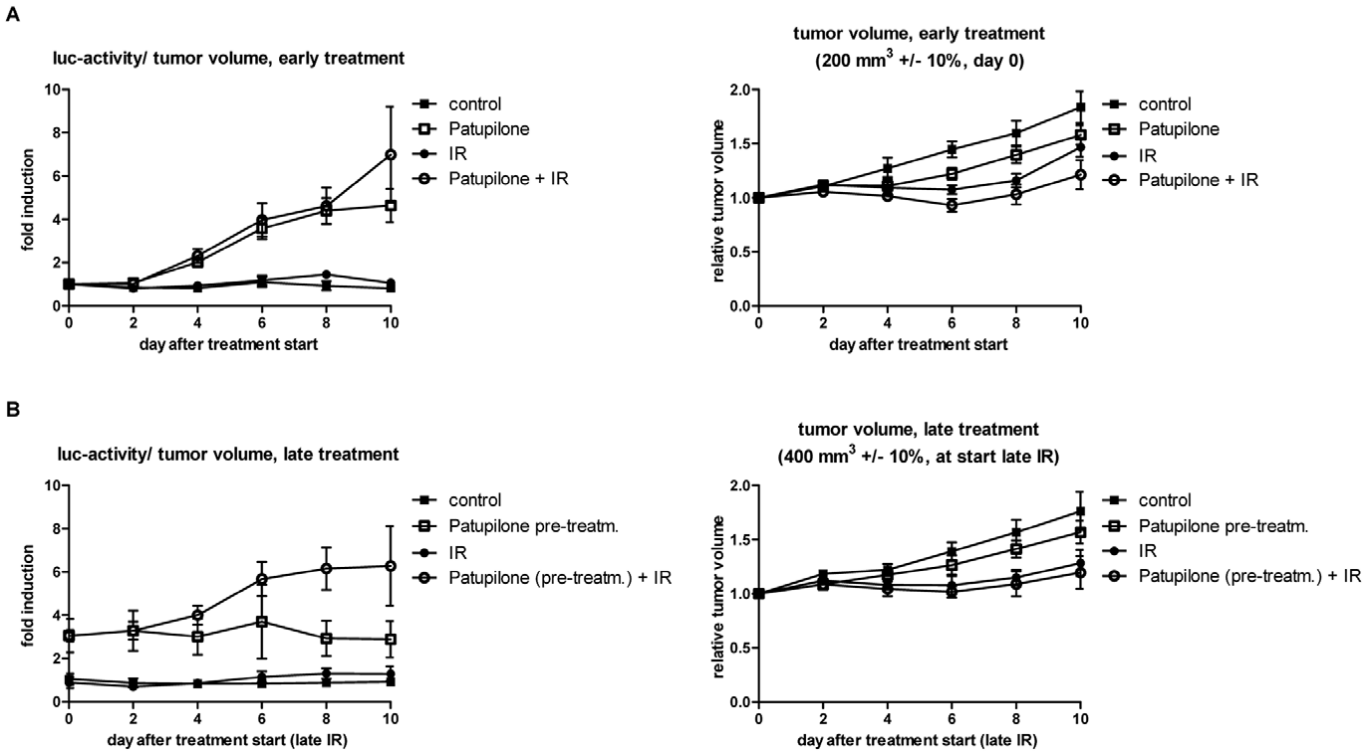
Tumor hypoxia in response to the combination of patupilone and ionizing radiation. (A) Luciferase activity and tumor growth of A549 ODD-Luc-derived xenografts in response to patupilone (2 mg/kg, day 0) and ionizing radiation (3×1 Gy, day 1–3) alone and in combination, treated at a tumor volume of 200 mm^3^±10%. (n_control_ = 7; n_IR_ = 5; n_patupilone_ = 19; n_combined_ = 5) (B) Luciferase activity and tumor growth of A549 ODD-Luc-derived xenografts in control and patupilone-pretreated mice. Irradiation was performed at a tumor volume of 400 mm^3^±10% (control) or 10 days after patupilone-pretreatment, (n_control_ = 7; n_IR_ = 5; n_patupilone_ = 6; n_combined_ = 5). Data used in Figure 4 and 5 are replotted into this figure for better comparison of the treatment groups. Error bars represent mean ±SE of fold induction luciferase activity/tumor volume or relative tumor volume per group.

First, A549 ODD-Luc xenotransplanted mice at a small tumor volume (200 m^3^) were treated with patupilone (2 mg/kg) and ionizing radiation (3×1 Gy) as part of a concomitant treatment regimen. In comparison to the intermediate treatment response to IR and patupilone alone (see above), tumor growth was further suppressed in response to the combined, concomitant treatment modality already within this short observation period. Interestingly, induction of luciferase activity in response to the combined treatment modality was similar to the increase in luciferase activity after patupilone-treatment alone and thus dominated over the effect of luciferase activity after irradiation (Figure 6A).

Next, A549-ODD-Luc xenotransplants were treated with placebo or patupilone alone (2 mg/kg) at a small tumor volume (200 mm^3^) and were adjuvantly irradiated once the tumors reached a volume of 400 mm^3^+/−10% (in control and patupilone-pretreated mice, which corresponds to the day of highest patupilone-induced luciferase activity). Irradiation of placebo-treated mice only resulted in a slight induction of luciferase-activity, while irradiation of patupilone-pretreated mice further enhanced the already elevated luciferase-activity twofold. Interestingly though, the IR-induced tumor growth delay was similar in the placebo- and the patupilone-pretreated mice, despite the elevated hypoxic status in the patupilone-pretreated tumors at the time point of irradiation (Figure 6B). A differential tumor growth delay between the two groups was also not revealed at extended measurement times, during which the elevated luciferase activity again decreased. Interestingly though, *in vitro* clonogenic survival assays indicated that a prolonged pretreatment sensitizes to ionizing radiation (Figure S3, S4).

## Discussion

Radiosensitivity is progressively reduced when the pO_2_ in a tumor is below 15–20 mmHg. PO_2_ determination with invasive pO_2_ electrodes revealed a strong correlation between low pO_2_ prior treatment (<5–10 mmHg) and locoregional control after radiotherapy in squamous cell cancer of the head and neck, uterine cervix and non-small cell lung carcinoma [42], [43], [44], [45], [46]. However, the dynamics of tumor microenvironmental parameters like hypoxia during treatment might even represent the better prognostic factor with regard to clinical outcome [47]. Furthermore tumor microenvironment-interfering agents might also negatively change tumor oxygenation and the hypoxic tumor fraction by itself, thereby affecting the treatment response to irradiation. Thus, a combined treatment modality might be challenged by new potential hazards created by the combination itself [3], [6]. To address and investigate these processes and endpoints, non-invasive methods are required that allow serial determination of tumor hypoxia.

Here we established a non-invasive luciferase-based reporter system to serially probe tumor hypoxia, and determined the course of treatment-induced changes in tumor hypoxia in response to ionizing radiation and the clinically relevant microtubule stabilizing agent patupilone. The fusion construct luciferase, linked to a minimal ODD-domain, was stably expressed in tumor cells, but rapidly degraded under normoxic conditions by the oxygen-sensing prolyl hydroxylases. This hypoxia-sensing approach was previously developed in mouse models, which express this fusion protein ubiquitously and even as an ODD-luciferase transgene in a spontaneous murine mammary carcinoma model [37], [48]. Here we demonstrate that this approach could also be easily adapted to tumor xenografts in order to probe the effect of clinically relevant antitumor treatment modalities on tumor hypoxia. In contrast to HRE-based-luciferase hypoxia-reporting systems, the ODD-luciferase system with a minimal ODD-domain has the advantage to be robust against putative interference of agents of interest with the signaling cascade upstream of HIF-1, thereby avoiding false positive or negative readouts. Control experiments on the expression level of prolyl hydroxylase 2 and 3 in the A549 tumor cell line and in histological A549 ODD-Luc cell-derived tumor sections did not reveal treatment-induced changes, which might have influenced the reporter system (data not shown).

Our *in vitro* experiments revealed that the ODD-luciferase hypoxia reporter systems can be used to sense and differentiate decreasing O_2_-levels even as low as 0.2% O_2_. Thus, this approach – at least in our cell system - is more sensitive than pO_2_-measurements with the hypoxia marker pimonidazole, which did not discriminate hypoxic milieus below 1.5% O_2_, corresponding to 10 mmHg, and more sensitive than 18F-fluoromisonidazole (FMISO), which is used as hypoxia tracer for positron emission tomography in experimental tumor models and which has similar chemical properties as pimonidazole [49]. While the ODD-luciferase reporter system is highly sensitive to detect tumor hypoxia, unfortunately, the resolution of the *in vivo* bioimaging system is rather low. Therefore, only collective luminescence derived from the luciferase activity in the whole tumor could be determined, representing the mean tumor oxygenation status in the whole tumor. Thus, we could not distinguish between tumor areas with high or low tumor hypoxia and could not identify tumor sections that are specifically prone to treatment-induced changes in tumor hypoxia. Future *in vivo* bioimaging approaches with improved resolution are required to overcome these drawbacks.

The dynamics of tumor hypoxia in tumor xenografts were probed in response to minimal fractionated irradiation and the microtubule stabilizing agent patupilone. Independent of the initial tumor volume, irradiation resulted in a transient tumor growth arrest with a slight increase in the hypoxic fraction (volume-corrected luciferase activity). A drop in the hypoxic tumor fraction 5 days after treatment end in tumors treated at a small tumor volume coincided with resumed tumor growth, which could be due to the recovery or normalization of the tumor vasculature. We previously demonstrated that the hypoxic fraction in response to low dose, fractionated irradiation changes only minimally, but that the tumor vasculature nevertheless may undergo structural changes (e.g. switch to intussusceptive angiogenesis) with full recovery only after extended tumor regrowth [3], [50].

Single treatment with patupilone resulted in extended tumor growth arrest in both tumor models (A549, HCT116) and a strong, prolonged increase in the hypoxic tumor fraction. This correlation was accidentally confirmed in three (out of nineteen) patupilone-non-responding animals on the level of tumor growth and tumor hypoxia. No substantial patupilone-induced tumor growth delay was observed in these three patupilone-treated xenografted mice, and likewise, luciferase activity did not increase in these tumor xenografts. We could not explain the lack of responsiveness in these three mice, but more importantly they illustrate the correlation between the anti-tumoral effect of patupilone and the increase in intratumoral hypoxia in response to patupilone treatment (data not shown).

Thus, an increase in tumor hypoxia could represent an early surrogate marker for treatment sensitivity towards patupilone and likewise towards other microtubule stabilizing agents. However, we have only limited mechanistic insights how patupilone affects tumor hypoxia. Patupilone has anti-angiogenic and vascular disruptive activity leading to reduced tumor blood volume, which finally could translate into enhanced tumor hypoxia [51]. Patupilone directly interferes with HIF-signaling, which eventually results in a diminished tumor hypoxia stress response. Using a genetically defined patupilone-sensitive and -resistant tumor model, we previously demonstrated that the major cytotoxic insult occurs on the tumor cell level, and that the anti-angiogenic effect of patupilone required patupilone-dependent blockage of pro-angiogenic cytokine expression and secretion from the targeted, patupilone-sensitive tumor cell, such as VEGF [6], [38]. Here, histologic analysis of patupilone-treated tumors did not reveal a significant additional increase in pimonidazole-staining in comparison to the already intense pimonidazole-level for the A549 control tumors. This might be due to lack of correlation between HRE- or ODD-based bioimaging readouts and pimonidazole-staining, as already observed by others [27], [52], or the already low pO_2_-levels present in the control tumors, which does not allow anymore the use of pimonidazole to differentiate between treatment-dependent changes in pO_2_-levels (see above).

Own studies revealed a supra-additive antitumoral effect of patupilone in combination with ionizing radiation, against tumor xenografts derived from different tumor cell lines [5], [6], [41]. We previously demonstrated that ionizing radiation counteracted an inhibitor-of-angiogenesis-induced increase in tumor hypoxia in tumor xenografts when used as part of a combined treatment modality and thereby counteracted the potential risk of enhanced radiation resistance by the inhibitor of angiogenesis [3]. Concomitant treatment of patupilone with ionizing radiation did not reduce the patupilone-dependent increase in tumor hypoxia. Nevertheless, combined treatment resulted in an enhanced tumor growth delay, which could be due to a synergistic insult to the tumor cell compartment and tumor vasculature. Interestingly, the hypoxic fraction in the patupilone-pretreated xenografts even increased after adjuvant irradiation, which might derive from persistent fragility of the tumor vasculature in patupilone-pretreated xenografts and subsequent further damage on irradiation. Nevertheless and despite the presence of an enhanced hypoxic tumor fraction in the patupilone pretreated xenografts, irradiation still induced a similar antitumoral effect in patupilone-pretreated xenografts as in naive xenografts with a lower hypoxic tumor fraction. A radiosensitizing level of patupilone might still be present at the time point of irradiation, and indeed, *in vitro* clonogenic survival assays indicated that only a prolonged preincubation of cells sensitizes for ionizing radiation (Figure S4). This could be due to an enhanced uptake of patupilone overtime to high concentrations of patupilone, which eventually could radiosensitize the cells. Thus, a patupilone-dependent increase of tumor hypoxia might indeed nullify a putative radioenhancing effect of patupilone, when irradiation is applied at a later time point. Furthermore, we cannot rule out that patupilone-enhanced tumor hypoxia might only become relevant for higher single doses of ionizing radiation or for more extended treatment schedules than the one used in our study. Future studies including tumor control endpoints could resolve this issue prior to clinical application.

Overall, our results obtained with the two anticancer agents patupilone and ionizing radiation demonstrate, that the ODD-luciferase reporter system is a highly convenient, non-invasive approach to serially probe the dynamics of tumor hypoxia in murine tumor xenograft models. Patupilone-induced increase in tumor hypoxia might be used as marker for patupilone sensitivity. Furthermore, scheduling-experiments of the treatment modality of patupilone in combination with ionizing radiation reveal that patupilone-induced tumor hypoxia does not enhance radiation resistance as part of a concomitant or neo-adjuvant treatment regimen.

## Supporting Information

Figure S1 (A) Initial luciferase activity of A549 ODD-Luc and A549 Luc-only-derived xenografts at a tumor volume of 200 mm^3^±10%. (B+C) Luciferase activity per tumor volume of individual A549 ODD-Luc mice treated with patupilone (2 mg/kg) at 200 mm^3^±10% (B) or the individual respective control mice (C).
(TIF)

Figure S2 (A+B) Luciferase activity in stably transfected A549 ODD-Luc and A549 Luc-only cells, incubated for 8 hours under normoxia or DMOG and irradiated 4 hours after addition of DMOG. Error bars represent mean ±SE. (C) Luciferase activity and tumor growth of HCT116 Luc-only derived xenografts in mice, irradiated (3×3 Gy within 24 hours) at a tumor volume of 350–580 mm^3^ at the day of treatment start (n = 5). Fold induction of luciferase activity per tumor volume (solid line) and relative tumor volume (dotted line) were set to 1 at day 0. Error bars represent mean ±SE of fold induction luciferase activity/tumor volume or relative tumor volume.
(TIF)

Figure S3 **Luciferase activity and tumor growth of A549 ODD-Luc-derived tumor xenografts in control and patupilone-pretreated mice over a time period of 18 days.** Irradiation was performed at a tumor volume of 400 mm^3^±10% (control) or 10 days after patupilone-pretreatment. (n_control_ = 7; n_IR_ = 5; n_patupilone_ = 6; n_combined_ = 5). Error bars represent mean ±SE of fold induction luciferase activity/tumor volume or relative tumor volume.
(TIF)

Figure S4 **Clonogenic cell survival after treatment with patupilone and IR of A549 ODD-Luc cells.** Cells were treated with placebo or patupilone for 18 hours followed by an immediate clonogenic survival assay in response to irradiation, or preincubated with patupilone for 18 hours followed by 7 days of cultivation in absence or presence of patupilone, followed by a delayed clonogenic survival assay in response to irradiation. Error bars represent mean ±SE.
(TIF)
