# Supplementary figures and images for: Dynamics of Tumor Hypoxia in Response to Patupilone and Ionizing Radiation
Source: PLoS One. 2012 Dec 10;7(12):e51476. doi: 10.1371/journal.pone.0051476 (PMC3519688; doi:10.1371/journal.pone.0051476)

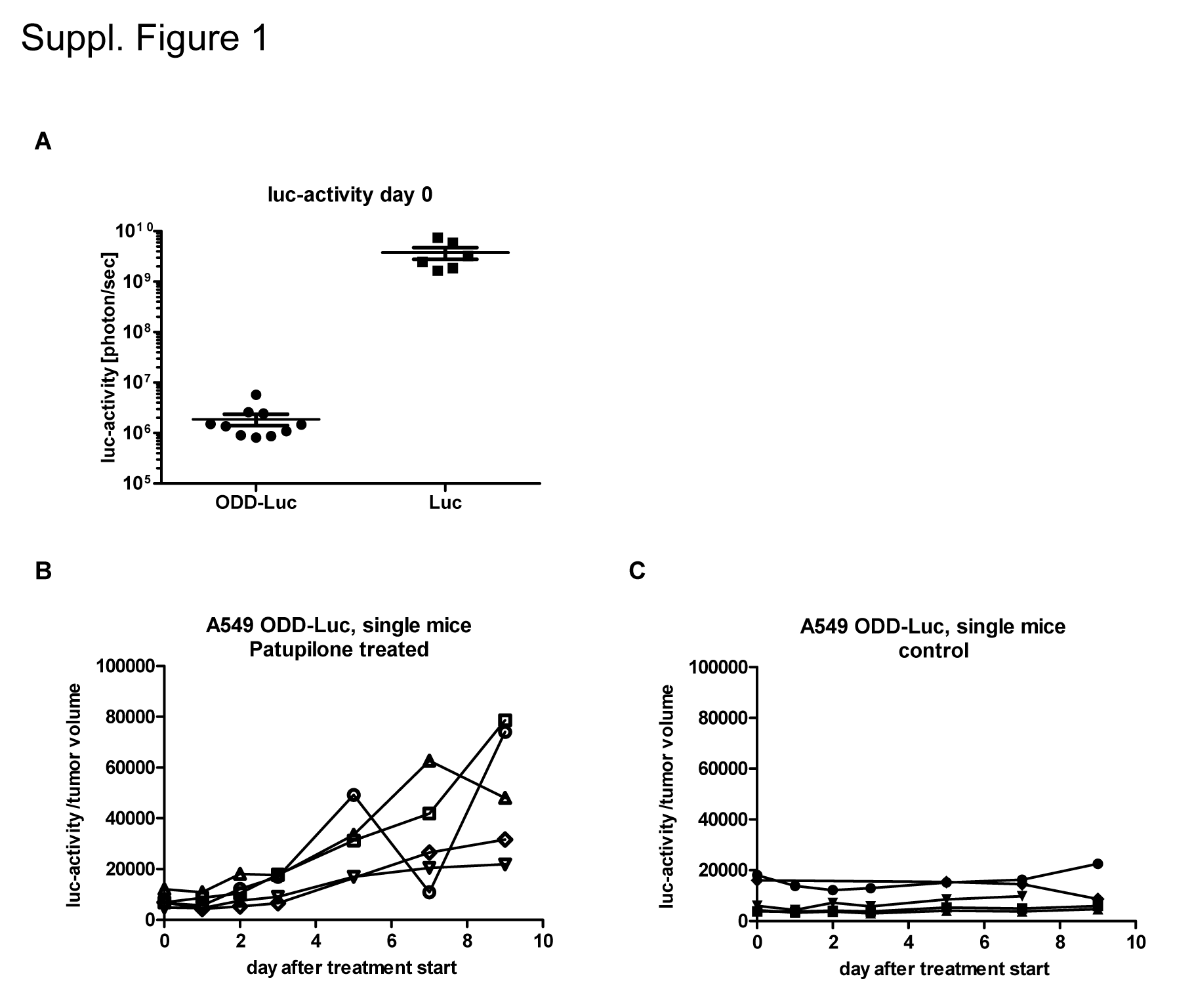

Supplement: Figure S1 — (A) Initial luciferase activity of A549 ODD-Luc and A549 Luc-only-derived xenografts at a tumor volume of 200 mm3±10%. (B+C) Luciferase activity per tumor volume of individual A549 ODD-Luc mice treated with patupilone (2 mg/kg) at 200 mm3±10% (B) or the individual respective control mice (C). (TIF) [file pone.0051476.s001.tif]

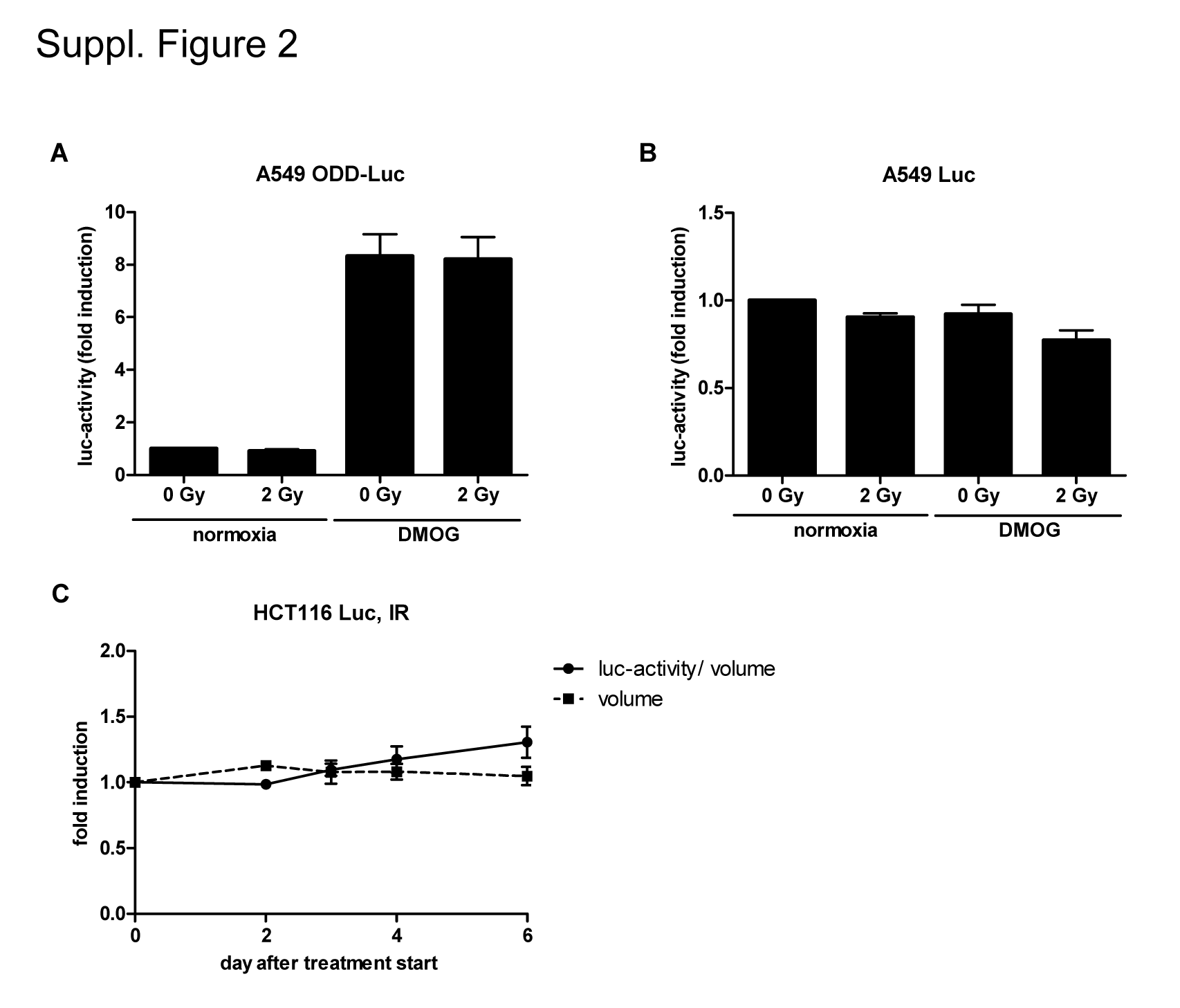

Supplement: Figure S2 — (A+B) Luciferase activity in stably transfected A549 ODD-Luc and A549 Luc-only cells, incubated for 8 hours under normoxia or DMOG and irradiated 4 hours after addition of DMOG. Error bars represent mean ±SE. (C) Luciferase activity and tumor growth of HCT116 Luc-only derived xenografts in mice, irradiated (3×3 Gy within 24 hours) at a tumor volume of 350–580 mm3 at the day of treatment start (n = 5). Fold induction of luciferase activity per tumor volume (solid line) and relative tumor volume (dotted line) were set to 1 at day 0. Error bars represent mean ±SE of fold induction luciferase activity/tumor volume or relative tumor volume. (TIF) [file pone.0051476.s002.tif]

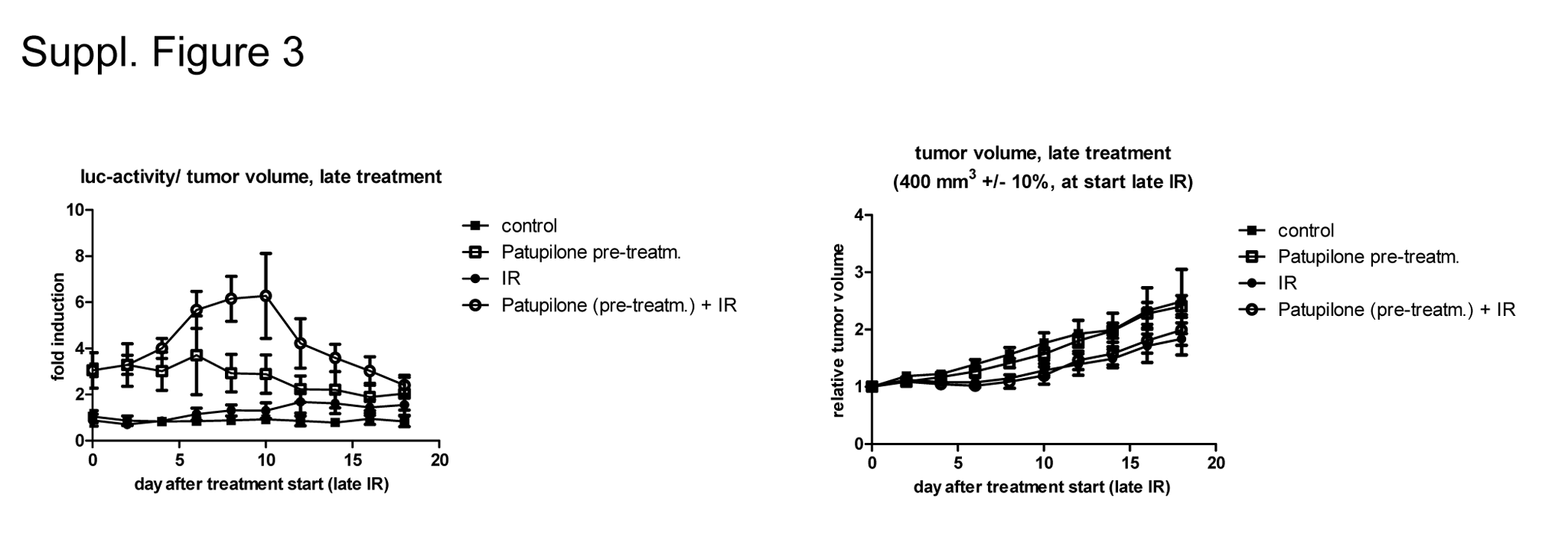

Supplement: Figure S3 — Luciferase activity and tumor growth of A549 ODD-Luc-derived tumor xenografts in control and patupilone-pretreated mice over a time period of 18 days. Irradiation was performed at a tumor volume of 400 mm3±10% (control) or 10 days after patupilone-pretreatment. (ncontrol = 7; nIR = 5; npatupilone = 6; ncombined = 5). Error bars represent mean ±SE of fold induction luciferase activity/tumor volume or relative tumor volume. (TIF) [file pone.0051476.s003.tif]

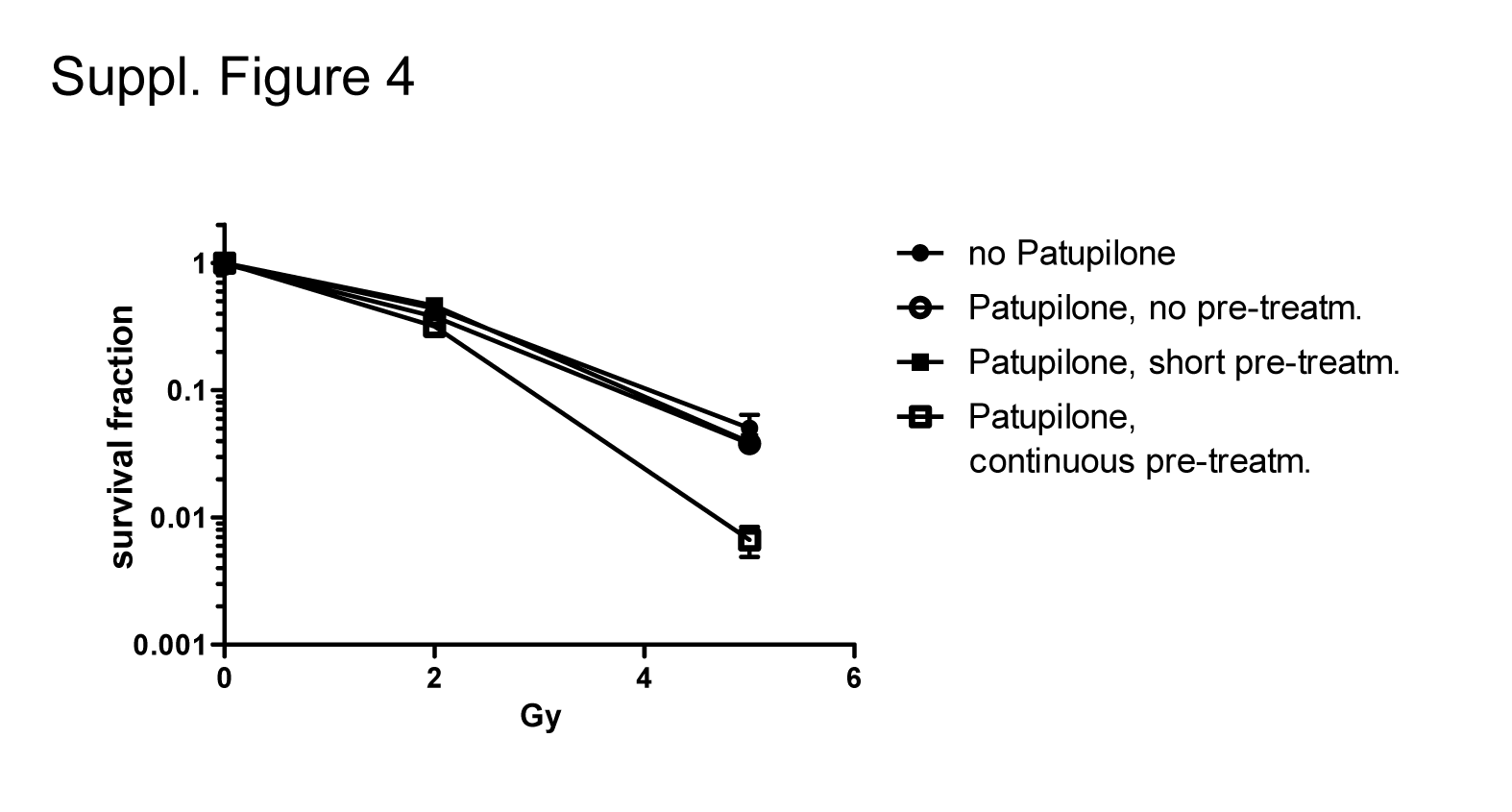

Supplement: Figure S4 — Clonogenic cell survival after treatment with patupilone and IR of A549 ODD-Luc cells. Cells were treated with placebo or patupilone for 18 hours followed by an immediate clonogenic survival assay in response to irradiation, or preincubated with patupilone for 18 hours followed by 7 days of cultivation in absence or presence of patupilone, followed by a delayed clonogenic survival assay in response to irradiation. Error bars represent mean ±SE. (TIF) [file pone.0051476.s004.tif]
